# The Selective Use of a Diverting Stoma in Rectal Surgery

**DOI:** 10.1007/s11605-022-05251-x

**Published:** 2022-02-01

**Authors:** Daitlin E. Huisman, Erik W. Ingwersen, Muriël Reudink, Boukje T. Bootsma, Gerrit D. Slooter, Jan Willem T. Dekker, Freek Daams

**Affiliations:** 1 Department of Surgery, Amsterdam University Medical Centers, Location VUmc, Amsterdam, The Netherlands; 2 Department of Surgery, Reinier de Graaf Gasthuis, Delft, The Netherlands; 3 Department of Surgery, Máxima Medical Center Veldhoven, Veldhoven, The Netherlands

**Keywords:** Anastomotic leakage, BMI Body mass index, ASA American Society of Anesthesiologists, TNM Tumour node metastasis, AJCC American Joint Committee on Cancer, AV Anal verge, MAP Mean arterial pressure

## Introduction

A diverting stoma is recommended by some as a routine procedure to lower incidence of AL and mitigate its consequences. Others, however, state that the diverting stoma is overused, as they describe no differences in AL rate between patients with and without a diverting stoma.^1^ Next to this, many patients experience stoma-related morbidity such as skin irritation, dehydration, stoma site complications, psychological distress, and reversal surgery with potential complications. Ultimately, many patients never undergo a reversal of their diverting stoma.^2^ Between 2007 and 2019, 89% of the patients who underwent rectal cancer surgery in Australia or New Zealand received a diverting stoma.^3^ In the Netherlands, however, the incidence of a diverting stoma in rectal surgery is considerably low (40% in 2016) and reduced in the last decade. ^4^ The relatively low application rate is possibly due to a selection of patients. This study was designed to identify patient characteristics and intraoperative conditions related to the presence of a diverting stoma and the impact on anastomotic leakage.

## Method

The data were used of a prospective, observational study from January 2016 to December 2019 from fourteen hospitals in four countries. This study was an additional subanalysis of the LekCheck study.^5^ All patients undergoing rectum resection with primary anastomosis were included.

## Results

A total of 351 patients were included for this sub-study. A diverting stoma was created in 97 patients (27.6%). The following seven variables were associated with a diverting stoma in univariate analysis: smoking status, neoadjuvant therapy, American Joint Committee on Cancer stage, tumor distance, fluid administration, blood loss, and an intraoperative event. In the multivariate analysis, the following variables were independently associated with a diverting stoma: tumor distance (*p* < 0.001), neoadjuvant therapy (*p* < 0.001), blood loss (*p* = 0.003), fluid administration (*p* = 0.003), and an intraoperative event (*p* = 0.022). Anastomotic leakage occurred in nine patients (9.3%) with a diverting stoma and in 34 patients (13.4%) without (*p* = 0.297). In patients with anastomotic leakage, fewer interventions were necessary when a diverting stoma was constructed (*p* = 0.001) (Tables 1, 2, and 3).

**Table 1:** Patient characteristics of patients with and without diverting stomas, compared in a univariate and multivariate analysis

					Univariate analysis			Multivariate analysis*		
Variable	Diverting stoma (*n* = 97)	No diverting stoma (*n* = 254)	Total (*n* = 351)	Missing	OR (95% CI)	*P* value		OR (95% CI)	*P* value	
Age (mean in years)	64.6 ± 11.0	65.8 ± 12.2	65.4 ± 11.9	*n* = *2*		0.289				
Sex						0.174				
Female	35 (36.1%)	112 (44.1%)	147 (41.9%)							
Male	62 (63.9%)	142 (55.9%)	204 (58.1%)							
BMI (mean in kg/m^2^)	26.3 ± 4.4	26.7 ± 4.4	26.6 ± 4.4	*n* = *5*		0.522				
ASA classification				*n* = *3*		0.552				
< 3	77 (80.2%)	209 (82.9%)	286 (82.2%)							
≥ 3	19 (19.8%)	43 (17.1%)	62 (17.8%)							
Diabetes mellitus				*n* = *2*		0.109				
No	80 (82.5%)	224 (88.9%)	304 (87.1%)							
Yes	17 (17.5%)	28 (11.1%)	45 (12.9%)							
Current smoker						**0.035**			0.131	
No		74 (80.4%)	228 (88.9%)		282 (87.5%)		1		1	
Yes		18 (18.6%)	26 (10.2%)		44 (12.5%)		2.00 (1.04–3.84)		1.81 (0.84–3.95)	
Pack years								0.603		
< 15 pack years		52 (53.6%)	144 (56.7%)		196 (55.8%)					
≥ 15 pack years		45 (46.4%)	110 (43.3%)		155 (44.2%)					
Alcohol use								0.257		
< 3 units per day		87 (89.7%)	216 (85.0%)		303 (86.3%)					
≥ 3 units per day		10 (10.3%)	38 (15.0%)		48 (13.7%)					
Disease						*n* = 1		0.224		
Malignant		92 (94.8%)	230 (90.6%)		322 (92.0%)					
Benign		5 (5.2%)	23 (9.4%)		28 (8.0%)					
Neoadjuvant therapy						*n* = 4		** < 0.001**		** < 0.001**
None		39 (40.6%)	179 (71.3)		218 (62.8%)				1	
5 × 5 radiotherapy		37 (38.5%)	53 (21.1%)		90 (25.9%)				3.86 (2.01–7.42)	
Chemotherapy		1 (1.0%)	2 (0.8%)		3 (0.9%)					
Chemoradiotherapy		19 (19.8%)	17 (6.8%)		36 (10.4%)					
AJCC stage						*n* = 32		**0.023**		0.613
I & II (T1-4N0M0)		28 (31.1)	103 (45.0%)		131(41.1%)		1		1	
III & IV (T1-4N1-2M0-1)		62 (68.9%)	126 (55.0%)		188 (58.9%)		1.81 (1.08–3.03)		0.84 (0.42–1.65)	
Tumor distance from AV								** < 0.001**		** < 0.001**
> 10 cm		46 (47.4%)	158 (68.7%)		192 (61.0%)	*n* = 36			1	
≤ 10 cm and > 5 cm		35 (41.2%)	61 (26.5%)		96 (30.5%)				2.46 (1.64–3.73)	
≤ 5 cm		16 (16.8%)	11 (4.8%)		27 (8.6%)					

*OR* odds ratio; *CI* confidence-interval; *BMI* body mass index; *ASA* American Society of Anesthesiology score; *AJCC* American Joint Committee of Cancer; *TNM* tumor node metastasis; *AV* anal verge; *cm* centimeter. Bold values are statistically significant (*p* < 0.05). Adjusted for: current smoker, neoadjuvant therapy, AJCC stage and tumor distance from AV

**Table 2:** Intraoperative factors of patients with and without diverting stomas, compared in a univariate and multivariate analysis

					Univariate analysis		Multivariate analysis*	
Variable	Diverting stoma (*n* = 97)	No diverting stoma (*n* = 254)	Total (*n* = 351)	Missing	OR (95% CI)	*P* value	OR (95% CI)	*P* value
Use of vasopressor						0.555		
No	67 (69.1%)	167 (65.7%)	234 (66.7%)					
Yes	30 (30.9%)	87 (34.3%)	117 (33.3%)					
Epidural use				*n* = 11		0.337		
No	55 (59.1%)	160 (64.8%)	215 (63.2%)					
Yes	38 (40.9%)	87 (35.2%)	125 (36.8%)					
Hemoglobin						0.363		
< 6.0 mmol/L female or < 6.5 mmol/L male	3 (3.1%)	4 (1.6%)	7 (2.0%)					
≥ 6.0 mmol/L female or ≥ 6.5 mmol/L male	94 (96.9%)	250 (98.4%)	344 (98.0%)					
Fluid administration						** < 0.001**		**0.003**
< 1000 mL	20 (20.6%)	104 (40.9%)	124 (35.3%)		1		1	
≥ 1000 mL	77 (79.4%)	150 (59.1%)	227 (64.7%)		2.67 (1.54–4.64)		2.50 (1.37–4.57)	
Blood loss						** < 0.001**		**0.003**
< 100 mL	28 (28.9%)	140 (55.1%)	168 (47.9%)		1		1	
≥ 100 mL	69 (71.1%)	114 (44.9%)	183 (52.1%)		3.03 (1.83–5.01)		2.34 (1.34–4.01)	
Intraoperative event				*n* = 6		**0.001**		**0.022**
No	78 (80.4%)	229 (92.3%)	307 (89.0%)		1		1	
Yes	19 (19.6%)	19 (7.7%)	38 (11.0%)		2.94 (1.48–5.83)		2.410 (1.13–5.13)	
Approach						0.797		
Open	9 (9.3%)	18 (7.3%)	27 (7.8%)	*n* = 6				
Laparoscopy	84 (86.6%)	218 (87.9%)	302 (87.5%)					
Laparoscopy with conversion	4 (4.1%)	12 (4.8%)	16 (4.6%)					

*OR* odds ratio; *CI* confidence-interval; *MAP* mean arterial pressure. Bold values are statistically significant (*p* < *0.05*). *Adjusted for: current smoker, neoadjuvant therapy, AJCC stage and tumor distance from AV

**Table 3:** Patients with and without a diverting stoma and occurrence of anastomotic leakage, days until leakage was detected and severity. Reinterventions were scored when Clavien-Dindo was grade 3 or higher

	Diverting stoma and AL (*n* = 9)	No diverting stoma and AL (*n* = 34)	Missing	*P* value
Anastomotic leakage	9	34		0.297*
Days until anastomotic leakage was detected			*n* = *5*	
< 7 days	4 (57.1%)	24 (77.4%)		0.257^
≥ 7 days	3 (42.9%)	7 (22.6%)		
Reintervention needed				**0.046^**
Yes	5 (55.6%)	30 (88.2%)		
No	4 (44.4%)	4 (11.8%)		
Death within 30 days postoperatively				0.370^
Yes	1 (11.1%)	1 (2.9%)		
No	8 (88.9%)	33 (97.1%)		

*AL* anastomotic leakage. *X^2^ test. ^Fisher’s exact test

Entries in boldface is due to the signifance *p* value

## Discussion

This study found that the following factors were independently associated with a diverting stoma: tumor distance, neoadjuvant therapy, blood loss during surgery, fluid administration, and an intraoperative event. The use of a diverting stoma in this study was relatively low (27.6%) and although the anastomotic leak rate was lower in patients with a diversion, this difference was not statistically significant. Other authors found that selective use of diverting stomas led to the same incidence of AL compared to policies in which diverting stoma was more routinely used.^6^ Proper application of selective use would drastically lower the burden of the stoma, preventing stoma-related complications (e.g., parastomal hernias, dehydration, stoma prolapse), discomfort, and costs, for many patients. On the other hand, it can potentially reduce complications in patients who are at high risk for AL, since fewer reinterventions were necessary for patients with AL and a diverting stoma.

## Conclusion

The study showed differences in patient characteristics and intraoperative variables in patients with and without a diverting stoma. A diverting stoma showed a protective effect as the impact of AL was less severe, resulting in fewer reinterventions. Selective use is therefore suggested, since it prevents unnecessary application while protecting patients. The current focus should be on techniques to identify patients with increased risk as soon as the rectum resection, in order to apply the protective stoma restrictively in patients at risk.
